# Central and Systemic Responses to Methionine-Induced Hyperhomocysteinemia in Mice

**DOI:** 10.1371/journal.pone.0105704

**Published:** 2014-08-25

**Authors:** Marina Mastelaro de Rezende, Vânia D’Almeida

**Affiliations:** Department of Psychobiology, Universidade Federal de São Paulo, São Paulo, Brazil; Universidade do Estado do Rio de Janeiro, Brazil

## Abstract

Hyperhomocysteinemia has been considered a risk factor for neuropsychiatric disorders, but the mechanisms involved in this process have not been completely elucidated. The aim of this study was to analyze the influence of hyperhomocysteinemia induction by methionine supplementation considering different levels and periods of exposure in mice. For this purpose, methionine supplementation at concentrations of 0.5 and 1% were administered in water to increase homocysteinemia in male C57BL/6 mice, and was maintained for 3 time periods (2, 4 and 6 months of treatment). The results from one-carbon metabolism parameters, brain-derived neurotrophic factor (BDNF) concentrations and behavioral evaluation were compared. The 0.5% supplementation was efficient in increasing plasma homocysteine levels after 2 and 6 months. The 1% supplementation, increased plasma homocysteine after 2, 4 and 6 months. Little influence was observed in cysteine and glutathione concentrations. Frontal cortex BDNF levels showed a lack of treatment influence in all periods; only the expected decrease due to increasing age was observed. Moreover, the only behavioral alteration observed using a novel object recognition task was that which was expected with increasing age. We found that responses to hyperhomocysteinemia varied based on how it was reached, and the length of toxicity. Moreover, hyperhomocysteinemia can affect the normal pattern of one carbon metabolism during age increase in mice. These findings allow the establishment of a reliable animal model for studies in this field.

## Introduction

Hyperhomocysteinemia (HHcy) has been considered a risk factor for a wide scope of disorders, causing bone development abnormalities [1], cardiovascular commitment [2], [3] and neuropsychiatric disorders, such as Alzheimer disease [4], schizophrenia [5], depression [6] and bipolar disorder [7]. Additionally, the influence of homocysteine (Hcy) and related molecules in events ranging from embryologic development [8], [9] to aging [10] demonstrates the relevance of research in this field.

The neurocognitive aspects of HHcy are not completely understood, but studies have been performed correlating Hcy or related molecules in the modulation of neurotransmitters and neural factors, such as dopamine [11], GABA [12], serotonin and others [13]. Moreover, other factors may also exert important effects, such as oxidative stress [14], [15], exercise [16] and epigenetic modifications [5].

More recently, brain-derived neurotrophic factor (BDNF) has been considered to be a possible target for HHcy influence [13], [16], although the mechanisms involved are not completely understood. Changes in the conversion of S-adenosyl methionine to S-adenosyl homocysteine or the imbalance in the redox status [13], [17] could also explain BDNF participation in HHcy toxicity. Considering the well-known oxidative imbalance due to HHcy [18], it is also possible that Hcy influence is indirect in BDNF levels [19].

Experimental HHcy models are important tools for investigating these possibilities, and they are of increasing importance considering that there are many ways to induce HHcy in animals [20]. It is possible that different metabolic resources can be used to avoid accumulation of Hcy in supplementation treatments, which could lead to different final results, and may or may not affect BDNF production and function resulting in different behavioral consequences.

Considering the above statements and the current knowledge about the cognitive damage caused by Hcy in homocystinuric patients and how it worsens over time, the aim of this study was to analyze the impact of Hcy increase over different periods in the one-carbon metabolic pathway, BDNF and behavioral responses. To that end, we used 2 supplementation doses to induce HHcy and analyzed the consequences of its maintenance through 3 periods: 2, 4 and 6 months.

## Materials and Methods

### 2.1. Subjects

Male C57BL/6 mice, 21 days old, were treated with different concentrations of methionine to increase plasma Hcy concentrations, mimicking a hyperhomocysteinemia condition. The animals were exposed to L-methionine at 0.5% (M05) and 1% (M1) concentrations in drinking water (w/w) [20]. The treatment persisted for 2, 4 and 6 months. Animals were housed in standard cages (2 – 5 per cage) in a temperature-controlled room (22±2°C) with a 12∶12 h light-dark cycle (light cycle starting at 8 a.m.), and food and water were provided *ad libitum*. Animals were exposed to the same standard chow: water was the only extra source of methionine. The Institutional Ethical Committee (Comitê de Ética em Pesquisa da Universidade Federal de São Paulo) approved this study (CEP: 2012/11), and the mice used were treated according to the guidelines in the Ethical and Practical Principles of the Use of Laboratory Animals [21]. During the treatment period, weight, naso-anal length and pelage conditions were analyzed to check the animals' welfare. The Lee index from body mass analysis was calculated using weight and naso-anal length [22].

During all treatment periods, animals without water supplementation were maintained in the same conditions, which comprised the control group (CT).

**Figure 1:**
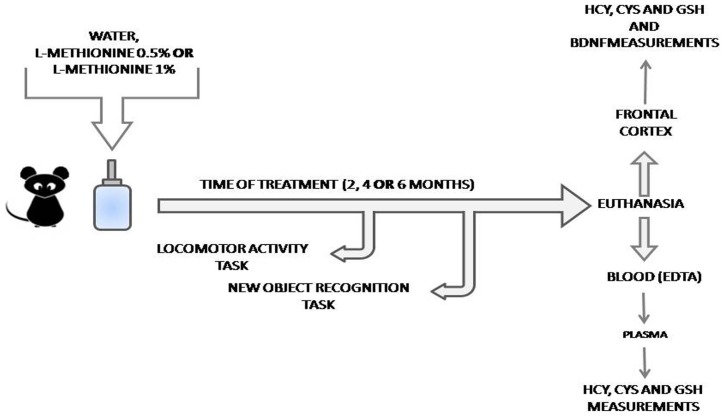
Representation of treatment and experimental procedures to which animals were exposed. (Hcy = Homocysteine; Cys = Cysteine; GSH = Glutathione; BDNF = Brain-derived neurotrophic factor).

After treatment and behavioral tasks, animals were euthanized by decapitation. Plasma was separated by centrifugation (10 minutes, 3000 rpm, 4°C) of blood collected in EDTA tubes, and the frontal cortex was dissected. All of the materials were stored at -80°C until analysis. A schema of the study is presented in figure 1.

### 2.2. Behavioral tasks

#### 2.2.1. Locomotor activity

To analyze baseline locomotor activity, animals were placed in Opto-Varimex cages (Columbus Instruments, Columbus, Ohio, 47.5×25.7×20.5 cm), similar to house cages, where interruptions of horizontal photoelectric beams were detected and interpreted as activity. Animals were placed in these cages for 1 hour, and locomotion events were counted each 5 minutes. This experiment always occurred between 4:00 h and 6:00 h (PM).

#### 2.2.2. New object recognition task

As a tool to assess memory, the ability of the mice to recognize a new object from a known one was analyzed. Animals were placed in a 20×20 cm empty square, white box for 5 minutes for habituation to decrease the animals' exploration of the new environment during the task [23]. The familiarization phase consisted of placing 2 objects at opposite corners of the box. For differentiation of the standard new object recognition task, distinct objects were used because object properties can influence the degree of animal exploration and discrimination [24]. One of the objects was a red metal heart-shaped object (object A), and another was a colorful wood rectangle (object B). During the test phase, object B was replaced by a magic cube-like object (object C). The familiarization and test phases lasted 8 minutes.

All objects were previously tested in an independent animal group and had similar indexes of preference and common properties, such as climbing and touching. The experimental box and objects were cleaned with a 10% ethanol solution after each phase of animal exposure. Between the habituation, familiarization and test phases, animals were returned to house cages and remained there for 10 minutes.

This procedure always occurred between 10:00am and 3:00pm, and the experiment was recorded by a video camera and subsequently analyzed by an experimenter that was blind to each animal's condition.

The analyzed parameters were as follows: object exploration time (time animal spent with its nose at least 0.5 cm from object), rearing (time animal spent in a standing position without leaning on the box's walls), leaning (time animal spent upright leaning against the wall) and grooming [25].

### 2.3. Biochemical measurements

#### 2.3.1. Plasma and frontal cortex homocysteine, cysteine and glutathione concentrations

Total plasma and frontal cortex homocysteine (Hcy), cysteine (Cys) and glutathione (GSH) concentrations were measured using HPLC (High Performance Liquid Chromatography) with an isocratic mobile phase and fluorescence detection at 385 nm excitation and 515 nm emission [26], [27].

For tissue quantification, homogenization with 1x phosphate-saline buffer (PBS) and protein precipitation with 100 g/L trichloroacetic acid with 1 mM EDTA was performed. A Shimadzu (Shimadzu Corporation, Kyoto, Japan) HPLC system coupled to a RF-10AXL fluorescence detector was used.

#### 2.3.2. Total protein quantification

Protein quantification of the frontal cortex was performed to adjust the results of Hcy, Cys and GSH using the Folin phenol reagent method [28].

#### 2.3.3. BDNF quantification

BDNF quantification was performed using the ELISA BDNF Emax ImmunoAssay System kit (Promega, Madison, Wisconsin) with an adaptation to the tissue lysis method used for protein extraction [29]. This adaptation of the protocol was required to fit the absorbance detection into the standard curve range, which corresponds to concentrations between 500 to 7.8 pg/mL. The obtained absorbance data were adjusted by the mass of tissue used.

### 2.4. Statistical Analysis

STATISTICA 7.0 software (Statsoft, Tulsa, Oklahoma) was used to perform data analysis. Before analysis of variance (ANOVA), quality tests were conducted to scan normality (Shapiro-Wilk's test) and homogeneity (Levene's test). In cases of repeated measurements, Mauchly's sphericity test was also performed. When required, standardization was performed using the Z-score.

The *Post-hoc* test used was Tukey, and p≤0.05 was considered statistically significant. Data are shown as the mean ± standard error of mean (SEM).

In the case of repeated measures (weight, length and Lee index), repeated measures ANOVA was used followed by the Newman-Keuls *post hoc* test.

To analyze the relationship between cortical Hcy and BDNF levels, a correlation test was performed.

## Results

### 3.1. Plasma Hcy, Cys and GSH

**Figure 2:**
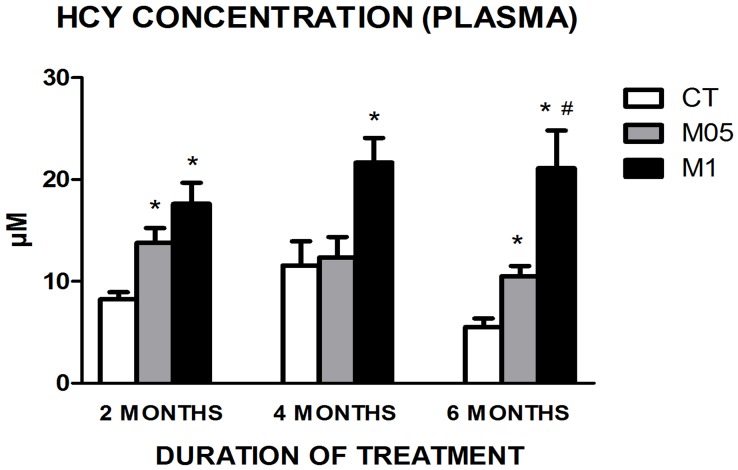
Plasma homocysteine concentration after 2, 4 and 6 months of treatment with water, methionine 0.5 and 1% solutions. (N = 9–11). CT = Control. M05 = Methionine 0.5%. M1 = Methionine 1%. * = *p*≤0.05 (Tukey's *post hoc* test) when compared with the CT group at the same period. # = *p*≤0.05 (Tukey's *post hoc* test) when compared with the M05 group at the same period. Data are presented as the mean ± SEM.

With respect to the plasma Hcy concentrations, the responses were dependent on the dose and duration of treatment. The results are shown in figure 2.

In this figure, we noted the treatment influence in each period, but changes over time between controls was also observed (data not presented in figure 2). A statistically significant reduction was observed in CT group between 4 and 6 months (*p* = 0.017), but this was not observed in the treated groups.

The cysteine and glutathione concentrations for the 3 groups of treatment duration are presented in table 1.

**Table 1:** Cysteine and glutathione (μM) concentrations in plasma after 2, 4 and 6 months of water or 0.5 and 1% methionine solution treatments.

		Cysteine (μM)	Glutathione (μM)
2 months	CT (N = 10)	354.21±17.5	181.44±17.7
	M05 (N = 10)	310.43±14.7	172.08±19.4
	M1 (N = 10)	297.69±11.2 *	192.36±16.0
*p* value (ANOVA)		0.029	0.724
4 months	CT (N = 10)	351.42±14.2	202.08±19.0
	M05 (N = 8–9)	317.29±12.4	269.15±28.1
	M1 (N = 9)	348.04±13.5	241.47±31.4
*p* value (ANOVA)		0.167	0.218
6 months	CT (N = 10)	268.54±13.7	257.95±19.7
	M05 (N = 9–11)	300.52±20.9	264.9± 31.7
	M1 (N = 10–11)	304.69±14.2	254.94±27.4
*p* value (ANOVA)		0.723	0.964

CT = Control. M05 = Methionine 0.5%. M1 = Methionine 1%. (N = 9–11). *p = 0.03 (Tukey's *post hoc* test) when compared with CT group in same period. Data are presented as the mean ± SEM.

Slight differences due to treatment could be observed in the plasma cysteine and glutathione concentrations in each period, beyond those which could be expected due to increased age. A reduction in the CT group cysteine concentrations could be observed, and the differences were significant between 2 and 6 months (*p* = 0.011) and between 4 and 6 months (*p* = 0.015). In the treated groups, the profiles were altered, M05 did not show any oscillation, and M1 showed an increase between 2 and 4 months (*p* = 0.031).

In respect of the GSH concentrations, no difference was observed in a treatment-dependent manner, but at different time points, the CT group showed an increase in its levels between 2 and 6 months (*p* = 0.021). The same profile was observed in the M05 group (*p* = 0.049), but no oscillation was detected in the M1 group.

### 3.2. Biometric parameters

**Figure 3:**
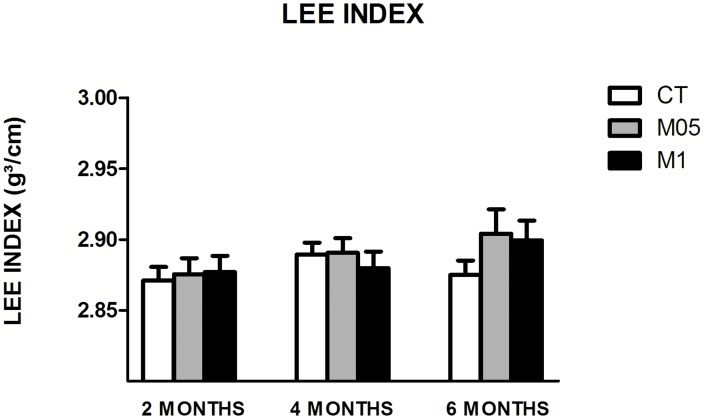
Lee index results over time (N = 10–22). CT = Control. M05 = Methionine 0.5%. M1 = Methionine 1%. Data presented comprise all animals at the specific time points. Data are presented as the mean ± SEM.

Although an increase in Hcy concentrations was achieved, it was not sufficient to interfere in bone development and animal growth. Comparisons of weight, length and the Lee index between groups did not result in significant differences. The Lee index results are presented in figure 3.

### 3.3. Frontal cortex Hcy, Cys and GSH concentrations

In relation to the Hcy concentration in the frontal cortex, a significant influence was observed just after 2 months of treatment in the M05 group, but this difference was not observed after 4 or 6 months.

The cortical concentrations of Cys and GSH were not significantly influenced by treatments in each period – 2, 4 or 6 months – at concentrations of either 0.5 or 1%. The data are shown in table 2.

**Table 2:** Frontal cortex homocysteine, cysteine and glutathione concentrations (nmol/mg protein) after 2, 4 and 6 months of hyperhomocysteinemia induced by methionine treatment.

		Hcy (10^−2^ nmol/mg protein)	Cys (nmol/mg protein)	GSH (nmol/mg protein)
2 months	CT (N = 8–10)	4.78±0.9	5.20±0.8	6.15±0.6
	M05 (N = 9)	10.03±1.1 *	5.39±0.4	6.73±0.4
	M1 (N = 6)	9.07±2.1	4.68±0.6	5.21±1.0
*p* value (ANOVA)		0.020	0.763	0.324
4 months	CT (N = 9–10)	9.07±1.9	5.50±0.7	5.14±0.5
	M05 (N = 8)	10.30±2.2	6.54±0.7	4.13±0.5^#^
	M1 (N = 4–5)	12.06±3.4	6.40±1.2	5.53±0.6
*p* value (ANOVA)		0.480	0.582	0.323
6 months	CT (N = 3)	9.01±2.7	5.82±0.7	6.15±0.7
	M05 (N = 6)	11.73±2.3	7.13±0.7	4.71±0.7^#^
	M1 (N = 7)	10.50±2.0	6.67±0.3	5.94±0.3
*p* value (ANOVA)		0.967	0.239	0.152

CT = control; M05 = Methionine 0.5%; M1 = Methionine 1%; Hcy = Homocysteine; Cys = Cysteine; GSH = Glutathione. Values presented as the mean ± SEM. * = Different from CT group in respective time of treatment (Tukey's *post hoc* test, p = 0.02). ^#^ = Different from M05 at 2 months (p<0.05)

Although no overtime oscillations were observed in the Hcy and Cys concentrations in any group, the treatment with 0.5% methionine, when analyzed at different time points, appears to influence GSH concentrations, since this group showed higher levels after 2 months of treatment when compared to 4 (*p* = 0.005) and 6 months (*p* = 0.044). Interestingly, this difference was not observed in the CT or M1 groups.

### 3.4. BDNF quantification

**Figure 4:**
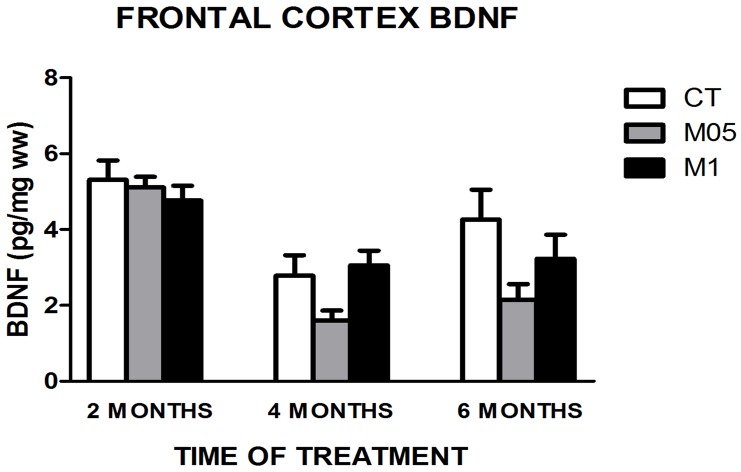
BDNF quantification in the frontal cortex (N = 6–10). CT = Control. M05 = Methionine 0.5%. M1 = Methionine 1%. The results are presented as the mean ± SEM.

Quantification of BDNF in the frontal cortex showed a lack of treatment influence in all periods, but in relation to increasing age, a diminution was observed between 2 and 4 months in the CT group (*p* = 0.011). In addition to the CT group, M1 showed a similar biochemical response (*p* = 0.041). The same pattern was observed in the M05 group, but an additional reduction could be observed between 2 and 6 months (*p* = 0.000 in both cases). The results are shown in figure 4.

### 3.5. Behavioral results

#### 3.5.1. Locomotor activity

In relation to treatment type and duration, no differences in locomotor activity between treated groups and CT were observed (data not shown). Furthermore, no oscillations over time were observed in any group.

#### 3.5.2. New object recognition

The new object recognition task was analyzed considering not only differential objects exploration, but also total object exploration, leaning, rearing and grooming to perform a more global analysis of animal behavior in this context [25].

With regards to total object exploration, both in familiarization and in the test, no differences related to treatment were observed. The results are shown in table 3.

**Table 3:** Total object exploration in familiarization and test phases (s) of new object recognition.

		Familiarization (s)	Test (s)
2 months	CT	119.00±10.9	122.89±9.9
	M05	110.50±8.2	109.10±6.6
	M1	99.00±14.0	107.44±12.0
*p* value (ANOVA)		0.463	0.475
4 months	CT	94.50±10.3	93.00±13.1
	M05	69.11±13.8	59.67±9.5
	M1	108.22±11.4	109.22±16.8
*p* value (ANOVA)		0.095	0.051
6 months	CT	72.00±8.2	62.00±7.4
	M05	71.90±11.5	68.00±8.0
	M1	63.73±12.3	60.3 ±10.0
*p* value (ANOVA)		0.812	0.766

CT = control; M05 = Methionine 0.5%; M1 = Methionine 1%. Values presented as the mean ± SEM.

In respect of the variation over time, during familiarization a significant reduction was observed in the CT group between 2 and 6 months (*p* = 0.008). Nevertheless, for the M05 group, a significant decrease was observed earlier, between 2 and 4 months (*p* = 0.042), although the difference falls between 2 and 6 months (*p* = 0.046). In the M1 group, a reduction was observed only between 4 and 6 months (*p* = 0.042).

In the test phase, the CT group showed a significant decrease between 2 and 6 months (*p* = 0.002), which also occurs for the M05 (*p* = 0.002) and M1 (*p* = 0.033) groups, but, as in the familiarization phase, M05 showed an earlier reduction, between 2 and 4 months (*p* = 0.001) and in M1, between 4 and 6 months (*p* = 0.027).

A preferential exploration could be observed in the test phase. The data are presented in table 4.

**Table 4:** Differential exploration of objects A and C in the test phase of new object recognition.

		Object A (%)	Object C (%)	T value	*p*
2 months	CT	40.61±7.7	59.39±7.7	5.485	0.000
	M05	35.70±10.0	64.30±10.0	6.375	0.000
	M1	33.95±9.1	66.05±9.1	7.507	0.000
4 months	CT	32.48±6.5	67.52±6.5	11.975	0.000
	M05	30.94±13.6	69.06±13.6	5.968	0.000
	M1	37.66±9.9	62.34±9.9	5.273	0.0 00
6 months	CT	28.69±8.6	71.31±8.6	10.460	0.000
	M05	35.05±8.1	64.95±8.1	8.644	0.000
	M1	31.20±10.8	68.80±10.8	5.510	0.000

CT = control; M05 = Methionine 0.5%; M1 = Methionine 1%. Values presented as the mean ±.

Preferential exploration of the new object in the test phase was expected, and treatment did not result in differences in respect of this parameter.

**Figure 5:**
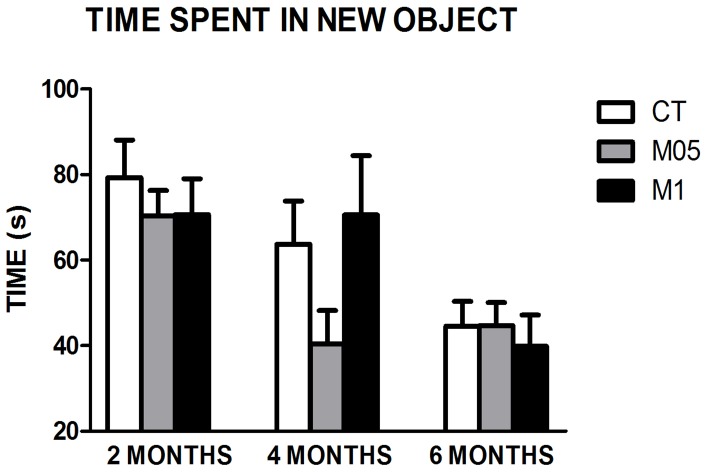
Time spent in object C (the new object) during the test phase of new object recognition. (N = 8–11). CT = Control. M05 = Methionine 0.5%. M1 = Methionine 1%. The results presented as the mean ± SEM.

The time spent in the new object exploration (object C) is presented in figure 5. No difference due to the treatment was found after 2, 4 or 6 months of treatment, but considering the duration of the treatment, a time-dependent reduction could be observed. In the CT group, there was a significant reduction between 2 and 6 months (*p* = 0.024), but it was different in experimental groups.

In the M05 group, reduction was observed earlier, between 2 and 4 months (*p* = 0.008), as well as the 2 and 6 reduction (*p* = 0.018). It is interesting to note that in the M1 group, no time-dependent influence could be observed (*p* = 0.050).

Secondary behavior analysis showed no treatment or duration influences in vertical exploration (leaning and rearing sum, data not shown) in the test phase, and when analyzing the ratio between grooming in the test *versus* familiarization phase, no treatment or duration influences were observed.

## Discussion

The plasma homocysteine concentrations presented by mice confirmed the effectiveness of treatments with methionine at both concentrations to induce HHcy (figure 2) even after only 2 months of treatment, but a lack of influence was observed after 4 months in the M05 group. This interesting finding suggests the possibility of a metabolic effort to address the high intake of methionine and return to basal levels with greater effectiveness with a lighter supplementation (0.5%) and shorter duration (4 months). Despite the same composition, the treatments used in this study had different influences in metabolism, most likely due to their concentration differences.

In addition to the treatment influence in each period, it is important to note that the animals showed changes in the homocysteine concentration profile over time. In the CT group, a significant decrease could be associated with the increase in animal age. This effect has already been described in rats by our group [30] and is the opposite of the effect reported in humans [31], [32]. Moreover, it is interesting to note that this decrease in Hcy concentrations over time was not observed in the treated groups, suggesting that an augmentation induced by methionine treatment in fact coped with the physiological decrease observed in aged animals. This result points to the possibility that the HHcy duration, in addition to concentrations reached over time, could contribute to the HHcy repercussions. Moreover, it is important to emphasize that there are a number of differences among humans and rodents concerning one-carbon metabolism [33] that could impede translational studies.

The treatments established in this study produced a small influence in plasma cysteine and glutathione concentrations. The lack of influence can be due to particularities in rodent metabolism, in which the transsulfuration pathway is highly activated [33] and glutathione is preferentially produced. It is important to note that apart from this slight influence, there were changes in the profile as a result of increased age which could play a significant role in the HHcy induction models.

Biometrical parameters were not influenced by treatments (figure 3), which could be due to the mild Hcy concentrations reached. It is important to note that in humans the alterations in these parameters are more related to higher levels of Hcy (e.g. in homocystinuria) than to HHcy in which plasma concentrations are set around 100µM [34].

Hcy concentrations in the frontal cortex showed differences compared to the control group after just 2 months of treatment with 0.5% methionine (Table 2). This result was not expected, mainly because a previous study [35] suggested that to reach tissue Hcy accumulation, longer supplementation is needed. However, when we observed the concentrations presented at each time point, the CT group at 2 months had lower values, that became higher at 4 and 6 months. Despite the natural reduction presented in circulating Hcy concentrations with the increase of age in mice (Figure 2), the cortical concentrations were higher and, most probably because of this, the methionine treatment in the two doses used was not sufficient to change these values.

It appears that the circulating and tissue Hcy concentrations have little correlation since in plasma we observed a reduction in control-aged mice and no changes in the brain. The differences in these concentrations in mice with increased age point to the possibility of distinct mechanisms being elicited to handle with intermediates of one carbon metabolism in different tissues. The requirement of brain protection from the toxic effects of this amino acid could lead to a more efficient transsulfuration pathway in rodents' nervous tissue to avoid Hcy accumulation, a mechanism which was maintained even during the aging process, favoring the formation of GSH (table 2), an antioxidant that has a protective role against reactive oxygen species [36] and in our study was not changed after treatment or age increase.

Comparing Hcy and BDNF concentrations in the frontal cortex, our results indicated that treatment had little influence. This is emphasized when the correlation test was performed, and no correlation between these variables was observed (*ρ* = 0.00). The lack of changes in the Hcy levels can be due to its metabolism particularities; in the case of BDNF, it is possible that its levels were restored in the time point analyzed. A recent study [17] shows reduced BDNF levels in the hippocampus of rats, which occurs after acute homocysteine treatment, but it is usually restored after a short period of time. Furthermore, it is possible that BDNF, taking into account its importance to life maintenance [37] and memory processes [38], most likely had protective pathways to avoid or minimize external influence.

Despite the little influence found, it is noteworthy that plasma Hcy levels and neurotrophic factors seem to be related [39], but methodologies vary widely between studies, making results difficult to compare. More expressive results are usually observed in knockout models [40] in which there are significant alterations in various parameters and, also, a severe HHcy is found.

Concerning the behavioral results, we found no differences in locomotor activity evaluated in the activity box. This result was important because it served as a control for the new object recognition test. Differential activity and exploration could be a bias in new object recognition data interpretation, which was avoided by the observed locomotor performance. Despite the fact that we observed no differences in locomotor activity, a relationship between plasma Hcy and exploration behavior has been previously described [40], but in knockout animals (*Mthfr−/−)*, which have more expressive Hcy influence than in our study. Even considering dietetic manipulations, different responses were observed. In the B-vitamin-deficient diet locomotor alterations were observed, despite there being no memory influence [41]. Once again, it is important to highlight how manipulation influences all results concerning HHcy, since different concentrations and exposition periods were reached.

It is interesting to notice that besides the increase in locomotor and exploratory behaviors observed in animals with HHcy, exercise combined with enriched environment applied to cystathionine beta-synthase deficient mice (*Cbs +/−*) restored molecular mechanisms responsible for memory impairment and increases BDNF levels [16].

For exploratory activity, there were no differences observed in the new object test related to treatment in the familiarization and test phases. These data show that the animals spent the same amount of time exploring objects. Analyzing the time spent in total object exploration, it should be noted that considering the profile over time, treated animals presented changes, especially the M05 group. This group showed a significant reduction in exploration earlier than the CT group in both phases. These data suggest that low performance in this parameter should be anticipated after treatment, highlighting a treatment influence, manly in the M05 group. It could be due to the marked reduction in BDNF levels after 4 months of treatment, emphasizing the different responses to treatment. In relation to differential exploration between objects in the test phase, an early decrease in object C exploration was observed in the M05 group, also suggesting that low performance in this parameter should be anticipated.

Muehlmann and co-workers [42] suggested that stereotyped behavior, such as grooming, rearing and leaning, could be indicative of anhedonic or depressive behaviors in animals. Furthermore, considering the number of studies correlating Hcy and neuropsychiatric disorders, such as depression in humans [6], murine depressive-like responses [43] and, anhedonic behavior [44], one could expect changes in the object exploration test, but the findings of this study did not corroborate the behavioral effects of HHcy.

In this study, three different treatment protocols to induce HHcy were used and produced significant peripheral changes in the methionine pathway. The central effects of these changes presented by animals of different groups were less evident. The molecular mechanisms to explain these differences are under investigation.

## Conclusions

Dose and duration of treatment influenced methionine-induced hyperhomocysteinemia in different ways, mainly in respect of peripheral one-carbon metabolism consequences. In relation to central influences, it has less magnitude but was not negligible. One-carbon metabolism characteristics are important factors to be considered in hyperhomocysteinemia studies and could result in a number of consequences that, in turn, deserve attention in this research field.
